# Renal Cell Carcinoma as an Incidental Finding in Firefighters: A Case Series

**DOI:** 10.7759/cureus.9259

**Published:** 2020-07-18

**Authors:** Kyle W Geiger, Tanner J Wright, Levi Deters

**Affiliations:** 1 Medicine, Elson S. Floyd College of Medicine, Washington State University, Spokane, USA; 2 Urology, Spokane Urology, Spokane, USA

**Keywords:** renal cell carcinoma, firefighters, occupational medicine

## Abstract

The link between cancer, including cancers of the kidney, and occupational exposure in firefighters has been well established. Renal cell carcinoma has a tendency to present incidentally on imaging rather than with the classic symptoms of flank pain and hematuria. In this case series, we identify four firefighter patients, all of whom initially presented with a kidney tumor as an incidental finding. We examine the absence of other risk factors in these patients along with current screening guidelines. This report aims to detail how these tumors present incidentally as well as evaluate the current screening guidelines in an effort to build awareness within this population. Patient demographics, risk factors, length of firefighting career, final pathology, and postoperative recurrence were evaluated. Four males underwent successful partial or total nephrectomy. All who have had follow-up have been tumor free with renal function intact. None are dialysis dependent. The role of routine renal imaging of this population is explored.

## Introduction

In 2019, there were an estimated 73,820 cases and 14,770 deaths from cancers of the kidney and renal pelvis [1]. Of these cancers, renal cell carcinoma (RCC) is the most common. RCC typically presents in the sixth to eighth decade of life. One study cites a median age of 64 years with just 4.4% of patients presenting in the age range of 20 to 40 years [2]. It is established that firefighters are at increased risk for a number of types of cancer including cancers of the kidney [3-5]. This link is highlighted by occupational exposure to toxic substances [3]. Other documented risk factors include genetic predisposition/hereditary disorders, obesity, smoking, and various nephrotoxic industrial chemicals [6]. Routine screening may be indicated for populations with exposure and identifiable risk factors [7]. In this study, we examine a series of RCC among young firefighters in the Pacific Northwest who had a diagnosis of RCC after a tumor was found on imaging as an incidental finding. We examine the absence of other risk factors in these patients along with current screening guidelines. This report aims to describe how these tumors present incidentally as well as evaluate the current screening guidelines in an effort to build awareness within this population. 

## Case presentation

Materials and methods

A retrospective analysis was completed through our clinic’s electronic healthcare record. We searched for patients who were diagnosed with RCC with a history of a firefighting career. Records from 2014 to 2019 were analyzed. Four total patients were identified with a median age of 36.5 years at detection of tumor (mean 40.75, range 31-58). Demographics, risk factors, and imaging were gathered and imported into a Microsoft Excel file. A Qualtrics™ online survey tool was developed and used to gather information about length of firefighting career. This information is presented in Table 1.

**Table 1:** Demographics and Risk Factors BMI, body mass index

Case	Gender	Age	Length of fire career (years)	Smoking history	BMI
1	Male	38	18	None	28.1
2	Male	59	40	None	30.8
3	Male	35	19	None	30.4
4	Male	31	7.5	None	28.5

Case presentations

Case 1

**Figure 1:**
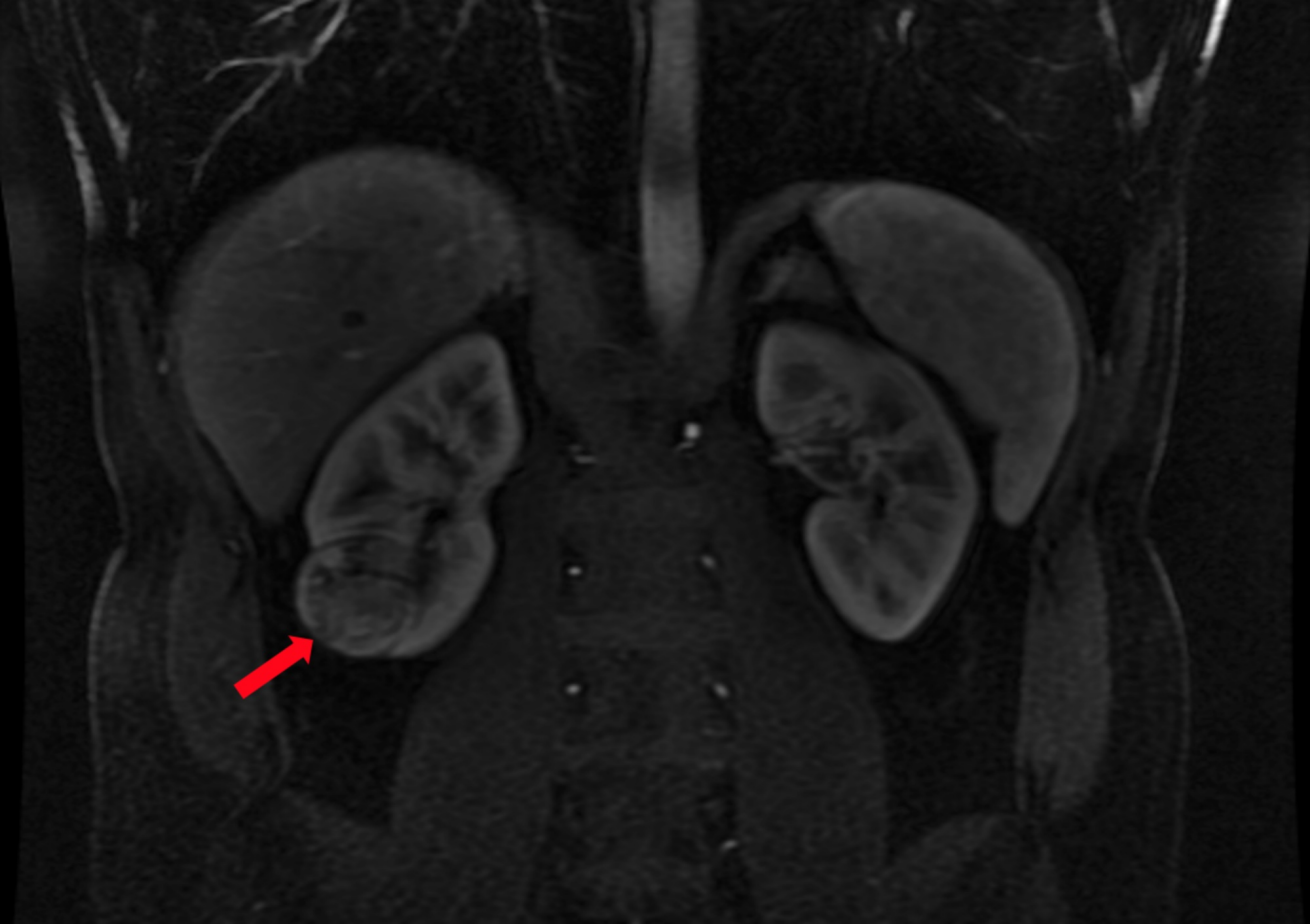
MRI showing image of right lower pole renal mass (red arrow)

A 38-year-old male with a history of chronic back pain underwent lumbar imaging. A 4-cm solid enhancing right lower pole renal mass was found incidentally (Figure 1). He was a professional firefighter for 18 years. His grandmother had kidney cancer at age 75 years. He denied gross hematuria and weight loss. The patient underwent robotic-assisted right partial nephrectomy. Pathology results showed T1a clear cell carcinoma with negative margins.

Case 2

**Figure 2:**
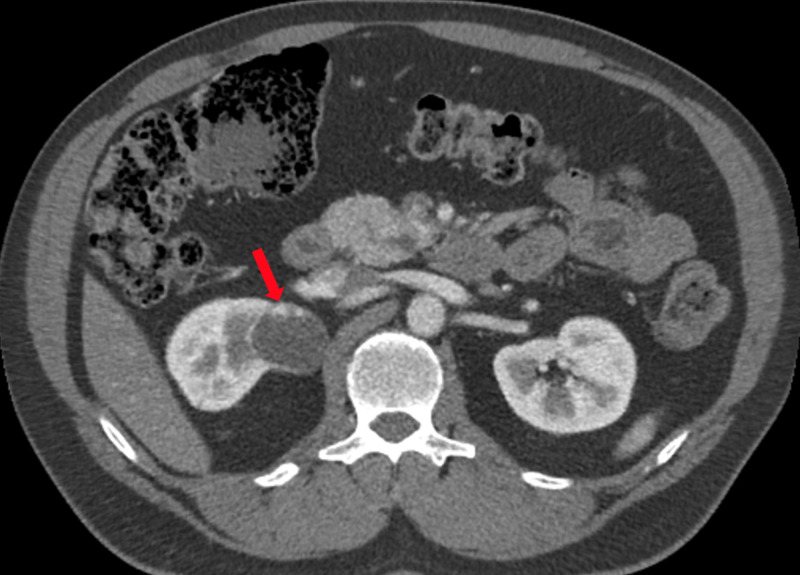
CT of the abdomen showing right renal mass (red arrow)

A 58-year-old male with a history of papillary mucinous neuroendocrine tumor of the pancreas was found to have a renal mass on imaging for pancreatic disease. He was a professional firefighter for over 40 years. He had no family history of renal cancer. He denied gross hematuria and weight loss. Imaging showed an approximately 4-cm cystic lesion with septations and some enhancing components (Figure 2). The patient underwent a robotic-assisted right partial nephrectomy. Pathology results showed T1b clear cell carcinoma with negative margins. CT at four-month follow-up showed no suspicions lesions. 

Case 3

A 35-year-old male who initially presented with calcium oxalate renal stones had a follow-up ultrasound that showed a 2.4-cm hyperechoic area in the left kidney. The patient then underwent MRI and was found to have a 3-cm mass. He had no family history of kidney cancer. He was a professional firefighter for 19 years. The patient underwent an open left partial nephrectomy. Pathology results showed T1a clear cell carcinoma with negative margins. CT at four-month follow-up showed no suspicions lesions. 

Case 4

**Figure 3:**
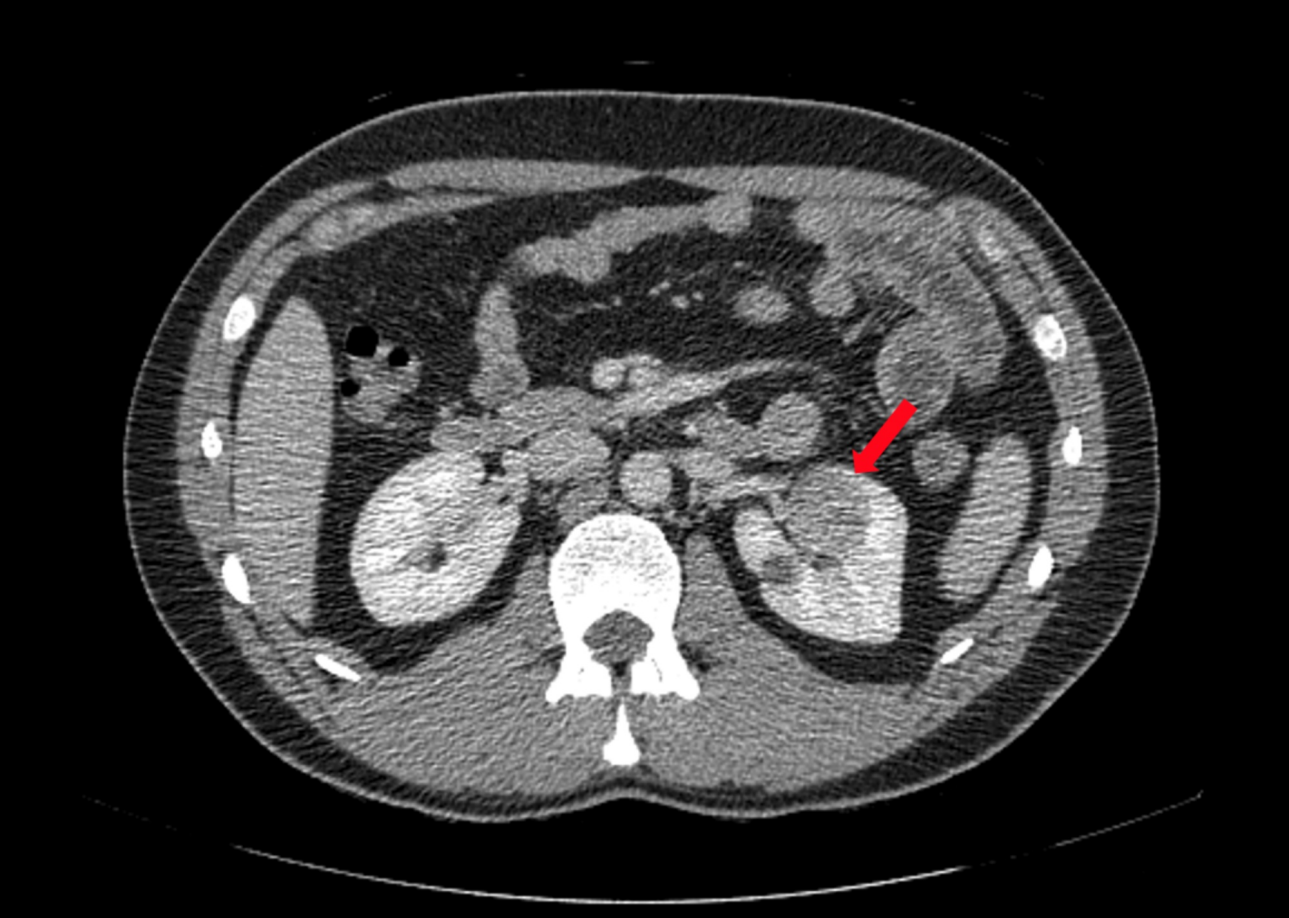
CT showing left renal mass (red arrow)

A 31-year-old male admitted from emergency department for gastroenteritis underwent CT with contrast showing a 3-cm enhancing left renal mass (Figure 3). He had no family history of kidney cancer. He denied gross hematuria and weight loss. He was a firefighter for seven and a half years. The patient underwent a laparoscopic left radical nephrectomy. Pathology results showed T1a clear cell carcinoma with negative margins. CT showed no suspicious lesions at seven months.

## Discussion

This small series shows examples of RCC in firefighters within our community. We detail the diagnosis of RCC via incidental findings and subsequent treatment in this population. We present four patients, without other obvious risk factors and/or clinical symptoms, who were diagnosed with RCC after imaging for reasons other than routine screening (back pain, nephrolithiasis, etc.). 

The literature has established that firefighters are at an increased risk for a variety of cancers including those of the kidney [3-5]. The mechanism for this increased risk is beyond the scope of our review, but is suspected to be due to exposure to carcinogens and possible genetic changes as a result [8]. RCC is one of the deadliest urological cancers. The Surveillance, Epidemiology, and End Results (SEER) database found an incidence-based mortality rate at 5.3% [9]. Recent studies within the firefighter population have recommended improving preventative measures [10]. Current screening methods and protocols for firefighters in our community are set by the National Fire Protection Association (NFPA) 1582: Standard on Comprehensive Occupational Medical Program for Fire Departments [11]. The NFPA recommends a yearly blood draw that screens for blood urea nitrogen (BUN), creatinine, and electrolyte abnormalities. They also recommend annual urinalysis for microscopic hematuria. However, renal function as measured by BUN, creatinine, and electrolyte abnormalities is not an established screening method for RCC. Other studies have found urinary dipstick is an inadequate test for RCC due to low sensitivity and specificity [12]. Renal ultrasound is an attractive option as a screening tool for RCC. Ultrasound is non-invasive, inexpensive, and widely used in the urological setting. A recent review of screening methods reports that ultrasound enables the detection of 85%-100% tumors of >3 cm in size, and 67%-82% of tumors 2-3 cm in size [7]. The same study reports ultrasound as less sensitive and specific compared to CT; however, we suggest this is outweighed by cost, convenience, and lack of radiation exposure.

All of our cases found renal masses incidentally, demonstrating a possible role for routine imaging. Other studies have found RCC is diagnosed incidentally over 50% of the time [12,13]. In addition, routine screening may be indicated for populations with exposure and identifiable risk factors such as firefighters [7]. Evaluation of routine screening methods for kidney cancer may be supported in this population. We suggest a larger scale study examining the cost-benefit of ultrasound screening for RCC in our firefighter population.

Our study does not examine epidemiological data due to a limited sample size. This case series only provides an example of these patients in one small clinic in the Pacific Northwest. Other studies are needed to examine this relationship in the broader Pacific Northwest and United States.

## Conclusions

Four of our firefighter patients were diagnosed with RCC via incidental findings on imaging. These cases highlight the possible need for additional screening in firefighters. There are limited studies that examine the role of routine renal imaging in this at risk population. We suggest a larger scale study examining the cost-benefit of ultrasound screening for RCC in the firefighter population.
